# Aneurysm and pseudoaneurysm of the left ventricle

**DOI:** 10.1016/j.amsu.2022.103405

**Published:** 2022-02-24

**Authors:** Jamal El ouazzani, Issam Jandou

**Affiliations:** a Department of Cardiology, Mohammed VI University Hospital Center, 60049, Oujda, Morocco; b Department of Urology, Ibn-Rochd University Hospital Center, Casablanca, Morocco; c Laboratory of Epidemiology, Faculty of Medicine and Pharmacy of Casablanca, Morocco

**Keywords:** Aneurysm of the left ventricle, Pseudo-aneurysm of the left ventricle, Myocardial infarction, Echocardiography

## Abstract

The severity of myocardial infarction lies in its complications. Certainly, there was a significant decrease in their impact thanks to the improvement of medical care and advent of early reperfusion methods, but there is still a considerable rate of complications that pose diagnostic and therapeutic problems. Among them, there are left ventricular aneurysm and pseudoaneurysm. These two complications are relatively rare, their diagnosis and treatment are often difficult. We have attempted to review the existing literature and discuss the characteristic findings of each entity.

## Highlights

• Aneurysm and pseudoaneurysm of the left ventricle are relatively rare, their diagnosis and treatment are often difficult.
• We have attempted to review the existing literature and discuss the characteristic findings of each entity.
• Despite the evolution of medical imaging, differentiation between these two pathologies remains difficult.

## Abbreviations

ACS: Acute coronary syndrome
ACE inhibitor: Angiotensin-converting–enzyme inhibitor
ARBs: Angiotensin II receptor antagonists
CT: computed tomography
LAD: Left anterior descending artery
LV: Left ventricular
LVA: Left ventricular aneurysm
MRI: Magnetic resonance imaging
MI: Myocardial infarction
NSAIDs: Nonsteroidal anti-inflammatory drug
STEMI: ST-segment elevation myocardial infarction
TTE: Transthoracic echocardiogram

## 1 Introduction

With the development of interventional cardiology, the occurrence of post-infarction ventricular aneurysms and pseudo-aneurysms has become rare. These complications must, however, be well known to the clinician, in particular because of their different evolutions (see Table 1).

**Table 1:** Comparative table between LV aneurysm and pseudoaneurysm.

	LV aneurysm	LV pseudoaneurysm
**Incidence**	8 à 15%/MI	Rare
**Physiopathology**	Expansion of the infarcted segments	Myocardial rupture in the pericardium
**Locations**	Anterior and apex	Posterior and lateral
**Risk of rupture**	+	+++
**Treatment**	Principally medical	Surgery
**Risque chirurgical**	Uncertain	Lower than medical treatment
**TTE diagnostic accuracy**	High	Low
**Reference imaging**	TTE±ventriculography, MRI +++ and cardiac CT scan +++.	Ventriculography, MRI+++, cardiac CT scan+++.
**Morphology:**		
1) Nack	Large	Narrow
2) Wall	Myocardium + pericardium±thrombus	Pericardium±thrombus.
**Complications**	- Heart failure.	
	- Ventricular arrhythmias.	
	- Thrombo-embolics.	
	- Rupture.	

## 2 Methodology

This review article is based on a literature search over a period of 3 months, using Google Scholar, PubMed, ScienceDirect, and Springer Link as search engines. We combined the terms aneurysm, pseudo-aneurysm, left ventricle, myocardial infarction, as either keywords or MeSH terms. The search was limited to English and French articles. The retrieved articles were reviewed for potentially relevant studies and reviews.

## 3 Left ventricular aneurysm

The left ventricular aneurysm (LVA) corresponds to a scar area in the form of a thin-pocket shape communicating with the rest of the LV by a wide necked losing its contractile function due to transmural necrosis. It is about a change in the shape of the necrotic wall and not of the extension of myocardial infarction (MI). Its incidence has clearly decreased after the advent of coronary reperfusion; it went from 38% to 15% even 8% of MI [1,2].

Left ventricular aneurysm most commonly results from acute myocardial infarction (MI). Other causes of LVA include Chagas’ disease, cardiac sarcoidosis, and presence of Lewitic gummas in the myocardium. Midventricular hypertrophy and obstruction have been associated with apical infarction and LVA [3]. Aneurysm of the membranous ventricular septum is an uncommon congenital anomaly that develops as a result of the spontaneous closure of a ventricular septal defect [4].

### 3.1 Pathogenesis of ischemic LV aneurysm

**Fig. 1:**
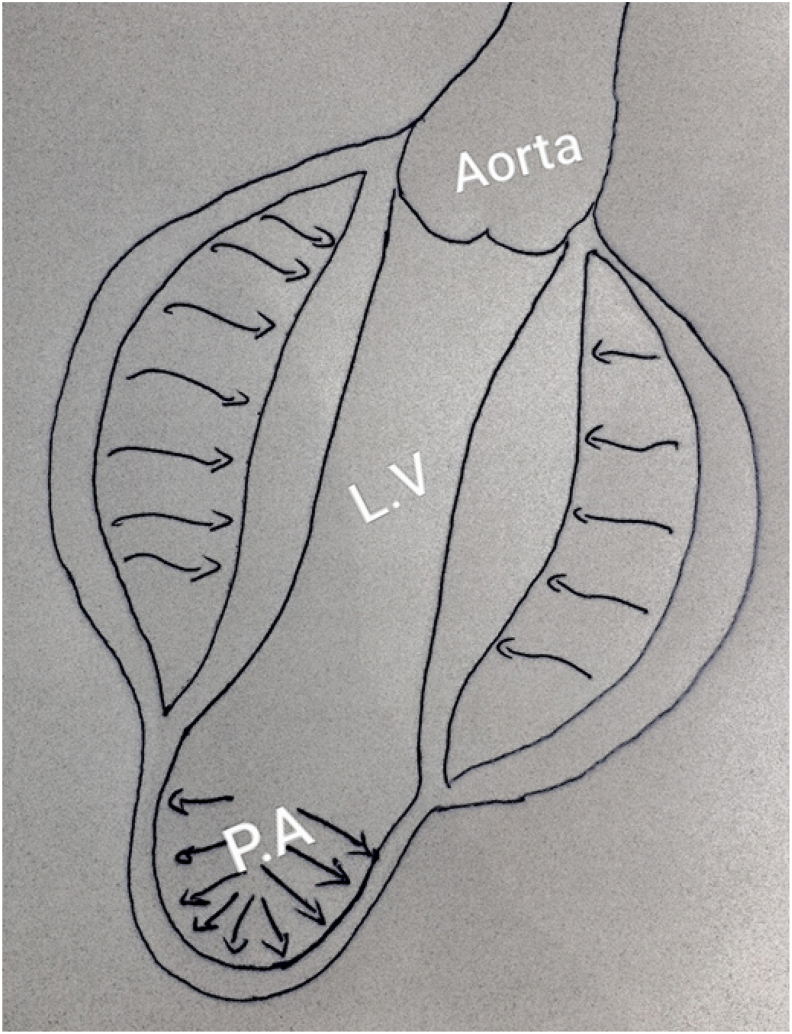
Left ventricular aneurysm: paradoxical systolic expansion of the fibrotic area.

True left ventricular aneurysm (LVA) probably occurs when the intraventricular pressure causes stretching of the infarcted, non-contractile, myocardial zone, which leads to the expansion of this relatively fragile zone, and thus the necrotic tissue swells out with each cardiac contraction (Fig. 1). Over time, the aneurysmal wall becomes fibrotic and continues to bulge out in systole and, resulting ineffective myocardial contractions and aggravates more the left ventricular function [5]. This complication occurs within days to weeks following ST-segment elevation myocardial infarction (STEMI), especially at the expense of the anterior wall secondary to left anterior descending artery (LAD) occlusion without an important collateral network, and rarely in case of triple vessel coronary artery disease when collaterals are well developed [5].

### 3.2 Diagnosis

LVA is often asymptomatic, and may be revealed either due to a complication or during a systematic transthoracic echocardiogram (TTE). Mitral regurgitation murmur, signs of left or right heart failure of and double auscultation area at the apex can be noted on the physical exam. The most suggestive electrical sign of LVA is the persistence of the elevated ST segment for more than 3 weeks after an acute myocardial infarction, which is stable in all records with necrosis Q waves in the same territory and T waves, whose amplitude are relatively weak in comparison with EGG complex, during acute myocardial infarction. The amplitude and the extent of the elevation do not correlate with the size of the aneurysm [7].

Chest X-ray may show a cardiomegaly, aneurysmal deformation of left–side arch of the heart, sometimes calcified aneurysm indicating its age.

**Fig. 2:**
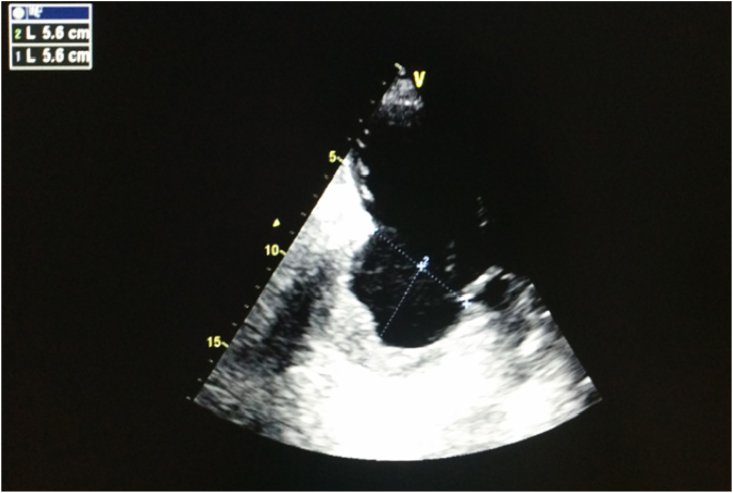
Echocardiographic image showing a chronic, thrombosed aneurysm of left ventricule inferolateral wall.

*Trans*-thoracic echocardiography has a high diagnostic performance in left ventricular aneurysm, with sensitivity and specificity exceeding 90% [8]. The apical views are the best to search and analyze LVA, on one hand because they allow a better visualization of the left ventricle and on the other hand because of the frequency of anterior and apical locations of the aneurysmal ectasia. It appears in the TTE as a thin-walled pocket developed in the territory of the MI communicating with LV by a wide neck (Fig. 2). This pocket deforms the LV cavity in diastole and animates a dyskinetic contraction in systole. The TTE must specify the location, size, shape of the aneurysm, neck size and identify the presence of intra-aneurysmal thrombosis or associated mitral regurgitation [8].

Visser et al. showed that the anterior topography is widely the most common with a percentage of 77% followed by the posterior location (17%) and then antero-posterior (5%) [9]. In another echocardiographic study, the same author had shown that the aneurysm occurs at the beginning of hospitalization for acute coronary syndrome (ACS) in nearly 50% cases, the remaining 50% occur during the first 3 months following the ACS. After 3 months its occurrence is exceptional [10].

Other imaging means may be needed in case of diagnostic doubt, namely magnetic resonance imaging (MRI), cardiac computed tomography (CT), and cardiac ventriculography. Cardiac CT, and cardiac MRI will have a higher diagnostic yield. Their high spatial resolution allow visualization of any plane of the heart and can thus show segments that are difficult to see on echocardiography. The tissue characterization of cardiac MRI make it ideal for evaluation of pseudoaneurysm of the ventricles and for distinguishing pseudoaneurysm from true aneurysms. The use of late gadolinium enhancement to identify the location and transmural extent of prior infarcts is particularly valuable [12].

### 3.3 Complications

The main complications are myocardial rupture, functional mitral regurgitation, aneurysmal thrombosis, heart failure and ventricular arrhythmias:Myocardial rupture is rare, because the wall of the aneurysm tends to fibroses, calcify and adhere strongly to the underlying pericardial becoming rigid. That is why it is said that the rupture of a chronic left ventricular aneurysm almost never occurs [5].

The mechanism of mitral regurgitation is variable according to the topography of MI. In case of anterior ectasia, it is secondary to left ventricular remodeling and deformity, in case of posterior ectasia, it is secondary to dysfunction of the subvalvular apparatus, in particular papillary muscles [2].

Heart failure is a function of the size of the LV residual cavity and its contractile reserves [2].

Intra-aneurysmal thrombosis occurs in 35%–40% of cases. It is secondary to the circulatory slowing in the aneurysmal sac, which explains that the thrombus is often wall-covering the bottom of the ectasia, rarely rounded or pedicled and mobile, and the low risk of thromboembolic accidents from chronic aneurysms because the clot is not very subject to turbulence in the blood stream [2].

### 3.4 Treatment

The early coronary désobstruction obtained under thrombolytics or by angioplasty has a preventive effect on the ventricular remodeling and consequently on the development of left ventricular aneurysm. The administration of an angiotensin-converting–enzyme inhibitor (ACE inhibitor) in the first 24 h after MI is particularly crucial in this situation because of its inhibitory effect of ventricular remodeling. Angiotensin II receptor antagonists (ARBs) and anti-aldosterones may also have similar effect [12]. Corticosteroids and nonsteroidal anti-inflammatory drug (NSAIDs) should be avoided during the first few days of MI, as they prevent myocardial healing and so promote aneurysm formation [13].

Surgical treatment consists in performing aneurysmectomy with myocardial reconstruction using a patch associated with coronary artery bypass grafting and correction of other mechanical complications. The goal of the surgery would be to eliminate the fibrotic tissue that causes ventricular arrhythmias, reduce the size of the left ventricle in order to decrease myocardial tissue oxygen requirements and to improve the contractile performance of the myocardium. This surgical treatment may be indicated mainly in cases of heart failure refractory to medical treatment, ventricular arrhythmias refractory to medical treatment and not accessible to ablation and in case of recurrent systemic embolism despite anticoagulant therapy [14,16]. The literature reports a perioperative mortality of 3–18% in case of isolated resection and 3–23% in case of an associated procedure, and a 5-year relative survival rate rarely exceeding 30%, but which remains higher than that of the equivalent population not surgically treated [15].

Anticoagulation of at least 6 months is indicated in any patient developing a mural thrombus during the first month of myocardial infarction [16]. This anticoagulation is indicated because systemic embolization can occur at 10% with documented wall thrombus and this risk appears to decrease with anticoagulant therapy [17,18]. Although the risk of embolization goes down considerably in the following months, treatment should be continued indefinitely in patients who do not have a significant risk of bleeding [6]. Patients developing left ventricular aneurysm without identifiable thrombi may also be anticoagulated because the post-mortem and perceptive incidence of thrombosis of the left ventricular aneurysm is at least 50% [17]. Long-term anticoagulation has little evidence and models practise are often different. Ga Yeon Lee et al. have shown that long-term anticoagulation with warfarin does not appear to affect the occurrence of cardiac and cerebrovascular, including systemic embolism [19].

## 4 Pseudoaneurysm of the left ventricle

Left ventricular pseudoaneurysm is a rare complication of myocardial infarction, it occurs when a rupture of the ventricular free wall is contained by overlying, adherent pericardium. Its spontaneous evolution is most often towards rupture with sudden death by tamponade [21]. The use of corticosteroids, NSAIDs and the presence of arterial hypertension may increase the risk of developing this complication [13].

### 4.1 Pathogenesis

A postmyocardial infarction left ventricle pseudoaneurysm occurs when pericardial adhesions contain a true rupture of the ventricular free wall [22].

### 4.2 Diagnosis

The Left ventricular pseudoaneurysm is often asymptomatic and the detection is often fortuitous during routine imaging or in post mortem. Progressive enlargement of the pseudoaneurysmal cavity may lead to the appearance of heart failure signs, some patients may present ventricular arrhythmias, others may develop thromboembolic complications following the thrombus expulsion from the aneurismal sac to the ventricular cavity [6]. The clinical examination may be normal or show signs of heart failure, sometimes a new murmur at auscultation [23]. The electrocardiographic signs and chest x-ray are similar to those of the LV aneurysm.

There are several available imaging modalities to make the diagnosis, including the TTE, the cardiac ventriculography, transesophageal echocardiography, cardiac MRI and the cardiac CT scan, but none is 100% accurate. TTE must be done first, since it is a non-invasive and available method, even though its diagnostic accuracy is low (about 26%) [24]. The pseudoaneurysm appears in the TTE as a pocket of varying sizes, sometimes expansive in systole that can contain thrombi or spontaneous echo contrast. In addition, it can occur at any location, but the posterior wall is the most common location, followed by the lateral, apical and the inferior wall, this can be explained by the protective role of the diaphragm on which rests the LV inferior wall [24]. In fact, several authors consider the neck size as the main differential element in imaging between the aneurysm and pseudoaneurysm; the neck is wide in true aneurysm and narrow in pseudoaneurysm.

**Fig. 3:**
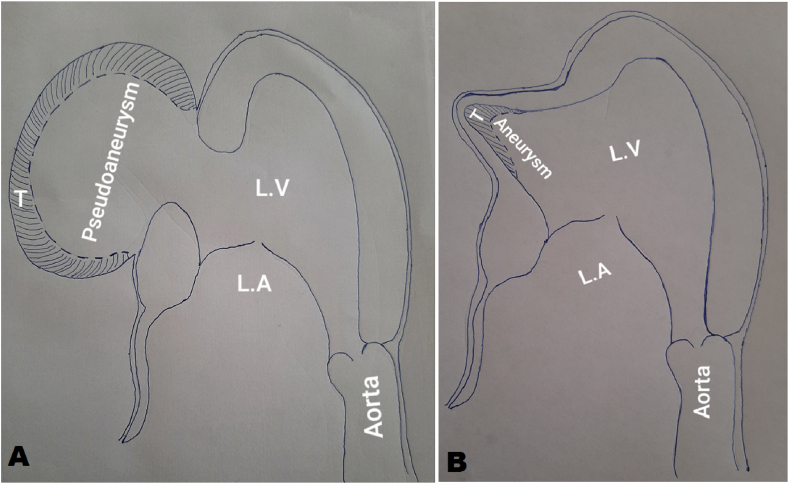
Morphological differences between the left ventricle aneurysm and pseudoaneurysm.

Ventriculography has a high diagnostic accuracy. It shows the pseudoaneurysm in the form of a cavity communicating to the LV with a narrow orifice. If a coronarography is performed concomitantly, there will be an interruption of coronary flow in the aneurysmal area (Fig. 3) [6].

In certain circumstances, where the diagnosis is difficult, cardiac MRI may show the presence or not of the myocardium in the aneurysmal wall, the thing that will make the difference between the LV aneurysm and the LV pseudo-aneurysm.

### 4.3 Treatement

Although some authors report series of non-operated patients with satisfactory survival rates [25,26]. Surgical treatment of left ventricular pseudo-aneurysm is urgently recommended whenever is possible because of the high risk of rupture (30–45%) and sudden death by tamponade [21,27].

## 5 Conclusion

Left ventricular aneurysm and pseudoaneurysm are a mechanical complications of MI that pose a problem of management. The surgical indication is difficult to carry and the operative result is not always brilliant. Prevention of remodeling is currently significantly improved by early revascularization techniques for myocardial infarction, as well as early prescription of ARBs, ACE inhibitors and anti-aldosterones.

## Provenance and peer review

Not commissioned, externally peer reviewed.

## Please state any conflicts of interest

All the authors have no conflict of interest neither financial nor ethical.

## Please state any sources of funding for your research

We have no funding for this work.

## Ethical approval

A copy of the written consent is available for review by the Editor-in-Chief of this journal on request.

## Consent

A copy of the written consent is available for review by the Editor-in-Chief of this journal on request”.

## Author contribution

Dr. IJ analysed and performed the literature research; Dr. EJ performed the examination and performed the scientific validation of the manuscript. Dr. El ouazzani Jamal was the major contributors to the writing of the manuscript. All authors read and approved the manuscript.

## Registration of research studies

1. Name of the registry:
2. Unique Identifying number or registration ID:
3. Hyperlink to your specific registration (must be publicly accessible and will be checked):

## Guarantor

Dr. El ouazzani Jamal.
